# Overcoming the translational roadblocks: a cancer care and research model

**DOI:** 10.1186/2001-1326-3-11

**Published:** 2014-05-09

**Authors:** Lauren Braun, Helena ML Daudt, Peter Watson

**Affiliations:** 1 Vancouver Island Centre, BC Cancer Agency, 2410 Lee Avenue, Victoria, BC V8R 6 V5, Canada

**Keywords:** Translational research, Roadblocks, Participatory research, Multidisciplinary

## Abstract

There are many challenges to the process of translating the knowledge gained in the laboratory into new clinical approaches that can meet the needs of patients, clinicians and the wider community. We describe here an initiative that has borrowed concepts and principles from participatory research to produce a new process embedded in a cancer center aiming to facilitate translational research and overcome the three translational roadblocks. The centre-wide project named Personal Response Determinants in Cancer Therapy (PREDICT) operates with the support of the centre’s leadership, staff, volunteers and patients to contribute to current and future cancer research successes. We describe the different phases of the project, the current structure and lessons learned during its evolution, highlighting how PREDICT contributes to translational research and its linkage to participatory research concepts. Despite the contextualized nature of the PREDICT initiative, we believe that the framework developed for the project has the potential to help other clinical centers to overcome the translational research roadblocks.

## Background

The pursuit of translational research has historically been dependent on collaboration and communication between disciplines. The “bench to bedside” concept, harnessing knowledge from basic sciences to produce “new approaches for prevention, diagnosis and treatment of diseases” in clinical settings [1] implies a connection between basic scientists and clinicians [2]. But the strength of this connection is sometimes tenuous and the US Institute of Medicine has identified two “translational blocks” in the undertaking of clinical research: (T1) “the transfer of new understandings of disease mechanisms gained in the laboratory into the development of new methods for diagnosis, therapy, and prevention and their first testing in humans” and (T2) “the translation of results from clinical studies into everyday clinical practice and health decision making” [1,3]. A third roadblock has more recently been defined: “the gap between knowledge need and discovery”: the integration between needs identified by patients, community, clinicians, governments and organizations and the work undertaken by scientists and researchers during the discovery process [4]. It has become a widely stated ‘dogma’ that there is an urgent need for more effective strategies for pursuing translational and clinical science [5-7].

Even though the challenges of translational research are widely recognized, conceptual frameworks and methods/tools to address these issues are still being developed [1,6-8]. Applying participatory research methods and principles to translational research has been identified as a promising approach to overcome the “translational roadblocks” [6]. Hebert et al. [8] argue that community-based participatory research, translational research and interdisciplinary research share both philosophical underpinnings and practical means for applying and sharing knowledge and techniques. Engaging multiple disciplines including academic and non-academic partners where each one contributes their expertise from the initiation of the research design phase can be beneficial to foster ownership, capacity building and empowerment of academic and non-academic partners [6,9]. Riley et al. [10] highlights that, although broad stakeholder engagement may seem counterintuitive as a strategy to speed research, investment in this area has the potential to improve recruitment, make the activity and the outcomes of research more relevant to more stakeholders and ultimately facilitate the adoption of research findings by practitioners [10].

In 2006, the British Columbia Cancer Agency – Vancouver Island Centre (BCCA - VIC) initiated a centre-wide project built on the ideas and energy of clinical and health care staff, borrowing principles of participatory research and aiming to address the challenges posed by expanding translational research. Details of the size and scope of this health care facility are available from our website (http://www.bccancer.bc.ca) but briefly, approximately 600 staff and volunteers at the center provide care and research opportunities to about 4,000 new patients each year. The project, endorsed by the centre’s leadership, created an environment where all staff, volunteers and patients contribute to current and future cancer research successes. This “Personal Response Determinants in Cancer Therapy” (PREDICT) project has contributed not only to the engagement of patients in the research process but also has transformed the way clinical research is conducted at the VIC. We describe here the PREDICT project as a model to overcome the three “translational blocks” by improving patient care and engaging staff and patients in research as part of routine clinical practice.

## Our model

### Design phase

In 2005, an opportunity to create a new model for the conduct of clinical research within which all staff and new patients could participate to improve future clinical care, was identified by a group of researchers at VIC and endorsed by the centre’s leadership. PREDICT was initially funded by a start-up grant from a government funded foundation which was matched by funding from a philanthropic foundation. The project was named ‘Personal Response Determinants In Cancer Therapy’ project (PREDICT) to convey a focus on enabling research focusing on aspects such as the tumour and its relation to treatment outcomes to encompass the patient-centered side of the cancer problem (i.e. patient factors such as biological, psychosocial and societal), by emphasizing the patient’s key role through participation, as part of a solution. The PREDICT model would evolve to have two components: a “permission to contact” mechanism, asking participants for permission to be contacted in the future for research; and a biobank component, collecting and storing blood samples of participants for future research. The research team, now re-named the PREDICT Steering Committee requested expressions of interest to all staff disciplines to join the PREDICT Design Team. The start-up grant provided backfill funding to the design team during the design phase. Ten staff members were selected to be members of the Design Team based on degree of experience working with patients, diversity of discipline or other qualities such as knowledge, ability and leadership that would facilitate an efficient team. Members of this team included a Change Management Consultant (who had been enrolled at the outset to assist with the organizational transformation), a Clinical Research Coordinator, a Research Phlebotomist, an Administrative Assistant, a Clerical Supervisor, a New Patient Clerk, a Nursing Supervisor, a Clerk, a Volunteer, a Registered Nurse and a Radiation Therapist. This team met for a two day workshop at the beginning of the project where they listened to overall goals envisaged by the steering committee, and then independently discussed the scope of design team, deliverables, and timelines as well as allocated responsibilities. The design team met weekly to bi-weekly as required from March to June 2006 and created an outline for the PREDICT process, making decisions about who would be approached, when and how to approach potential participants, consent, obtain a blood sample and re-contact patients after consenting. They sought feedback from colleagues and evaluated the process in the clinical setting by participating in a mock trial run.

Ethics approval for an initial pilot phase was obtained in November 2006. This approval followed an extended period of discussions around preliminary proposals with the University of British Columbia-BCCA Research Ethics Board (REB) that improved the final protocol design.

### Pilot study

In the project, new oncology patients are invited to participate at the time of their first appointment at the clinic. Patients who choose to enroll become research participants who agree to be contacted in the future by other specific studies related to cancer research (PREDICT’s “permission to contact” component) and also agree to the collection and storage of a blood sample (PREDICT’s biobank component). The project’s commitment is that this blood sample will be used for research into cancer, causes of cancer, cancer treatments or the effects of treatments. In January 2007, a four-month pilot study, was launched to test the feasibility, refine the processes, and document any potential difficulties of PREDICT. Two to four clinics per week were involved in this phase to identify and approach 100 eligible patients for their participation in PREDICT. Patients were asked to provide a blood sample to be stored and used for research (anonymised biological samples) as well as to grant a ‘permission to be contacted’ (PTC) by future research studies (PREDICT stores the BCCA registration number only in its database. This number allows the linkage to the Cancer Agency Information System, a robust and secure platform that stores demographics, clinical and treatment data of cancer patients). After further REB review, a second phase of the pilot was implemented in May 2007. Over the next 6 months, PREDICT was gradually expanded to all 20 clinics at the VIC. Table 1 summarizes the number of eligible and consented participants in phases 1 and 2.

**Table 1:** PREDICT accrual for phase I and II of the pilot stage

		**Eligible**	**Approached**	**Consented**	**Declined**	**Deferred**
Phase 1	n	104	97	93	3	1
	%	-	93%	96%	3%	1%
Phase 2	n	963	699	617	37	45
	%	-	73%	88%	5%	7%

Eligible: patients who meet the eligibility criteria; Approached: patients that were approached and offered PREDICT; Consented: patients that were offered PREDICT and consented to participate; Declined: patients that were offered PREDICT and did not consent to participate; Deferred: patients that chose to delay their decision.

As an opportunity for staff to become involved and engaged in the project, change management initiatives were implemented throughout the early phases of the project. For example; the involvement of care aids in the process of paging PREDICT personnel when an eligible patient is available to be approached has added efficiency to the consenting step. REB approval was obtained to proceed from the pilot phase in July 2008 and PREDICT has been operating as a full centre-wide program since this time.

### The PREDICT process

All new patients are potential participants of PREDICT. Potential participants are eligible if:

● They are a new patient to the BCCA-VIC.

● Their cancer diagnosis has been confirmed.

● They are 19 years of age or older.

● They fully understand the study and give their informed consent to participate as demonstrated by signing the consent form.

Potential participants are not eligible if:

● They are a new patient referred to the BCCA-VIC but do not have a confirmed diagnosis of pre-cancer (for example in-situ cancer) or invasive cancer.

● They have already started chemotherapy or radiation treatment for their in-situ or invasive cancer. If they are already on hormone therapy, they are still eligible.

● They are 18 years of age or younger.

● They are not competent to provide informed consent.

**Figure 1:**
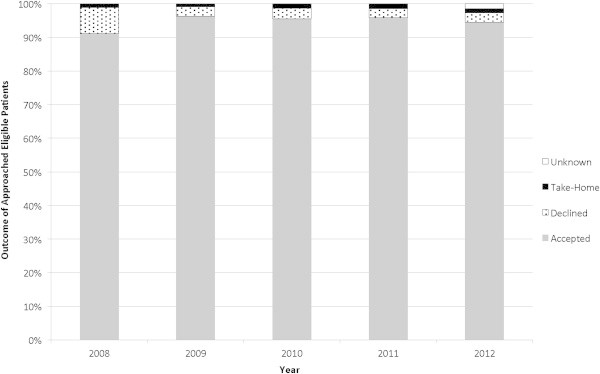
**Patient engagement in PREDICT remains high.** Accepted: patients that were offered and consented to participate in PREDICT; Declined: patients that were offered PREDICT and did not consent to participate; Take-home: patients that were offered PREDICT, have taken the consent home and have not consented or declined participation; Unknown: patients that were offered PREDICT, have not consented or declined or taken the consent home.

At the time of the first appointment at VIC patients are flagged and approached by a research intern for consenting. At the consenting interview they learn details about the project including the fact that their blood samples will be anonymised and that they will not receive information about study findings. Since the inception of PREDICT, there have been over 16,000 eligible new patients at the VIC and 53% (n = 8751) have been approached by PREDICT. The reasons for eligible patients not being approached are systematically recorded. The most common reason has been “health care decision” (63%), meaning that the physician or nurse deemed that it was not appropriate to approach the patient due to physical and/or psychosocial issues. Other reasons include “deferred/missed” (25%), “no show” (1%) and “other” (11%). Patient acceptability and engagement remains high with 95% of all approached eligible patients choosing to participate in the PREDICT project (Figure 1).

A series of standard operation procedures (SOPs) guide the clinical and laboratory activities. A governance structure was created to facilitate and ensure an ethically sound process to enable translational research. To facilitate the use of biospecimens and permission to contact collected by PREDICT, an Access Committee that accepts and reviews REB approved applications to use these data was formed and specific SOPs were created to guide governance and access/release of materials.

### The PREDICT governance

The PREDICT design has evolved with time to address the staff, patient and organizational needs. The current governance structure includes:

a) Management Committee, (7 individuals meeting 4–6 times per year) responsible for monitoring monthly accrual, personnel and general operations of the PREDICT project;

b) Operations Committee, (4 individuals meeting monthly) a sub-group of the Management committee responsible for running daily activities within the project;

c) Access Committee, (9 individuals meeting every time PREDICT receives an application) responsible for analyzing and approving or rejecting requests for sample and/or data release direct to PREDICT;

d) Oversight Committee, (14 individuals meeting 6–10 times per year) a VIC committee responsible for strategic decisions related to research at our centre and deployment of philanthropic support. PREDICT reports on major accomplishment and organizational changes to this committee 2–3 times a year.

With the involvement of these committees, PREDICT supports research conducted by (1) academics affiliated with the BC Cancer Agency and university institutions who are funded by academic sources and (2) academics sponsored and funded in whole or in part from industry or commercial sources. Access to PREDICT samples and data requires a formal application and approval process for all users. To date, 20 research projects have been supported by PREDICT. These include but are not limited to studies in the areas of Prostate Cancer, Breast Cancer, Colorectal Cancers, Melanoma, and Skin Cancer. The project relies on philanthropic support combined with partial cost recovery for operational expenses through user fees to maintain its activities.

Central to staff involvement in PREDICT is the involvement of a ‘Research intern’ to facilitate the consent process with potential participants: requesting permission to contact and blood specimen data. Research interns are trainee individuals who have already obtained a science undergraduate degree and are applying for higher training towards a career in a health-related discipline. Staff at all levels are directly involved in the oversight and training of these individuals within the clinic as they respond to referrals, and so contribute directly to the project. Thirteen individuals have been trained through the PREDICT project over the last 6 years: five are currently enrolled in medical school, four have completed/are enrolled in other professional schools (nursing, occupational therapy, education), two have pursued graduate studies and one is currently applying for dentistry school (we lost contact with one research intern).

As a way of fostering collaborative research partnerships and enhancing public trust in clinical and translational research, PREDICT reports on their activities regularly not only to the Oversight Committee but also to staff by presenting results and proposed organizational changes at staff meetings. We have recently developed an online newsletter, to be sent to staff twice a year as an additional aid to disseminate our successes and engage personnel. In addition, through our presence in the clinic, staff is reminded that there is an open communication channel between PREDICT operations and staff and many suggestions have been brought forward directly to members of the committee.

## Discussion

There are many challenges to the process of translating the knowledge gained in the laboratory into new clinical approaches that can meet the needs of patients, clinicians and the wider community. A key part of a strategic solution to overcome these challenges is to foster collaboration and communication between disciplines and between the public and scientists. We have described an initiative that has borrowed concepts and principles from participatory research to produce a new process embedded in a center that overcomes the three major road blocks to translational research: (T1) “the transfer of new understandings of disease mechanisms gained in the laboratory into the development of new methods for diagnosis, therapy, and prevention and their first testing in humans”; (T2) “the translation of results from clinical studies into everyday clinical practice and health decision making” - [1,3] and (T3) “the gap between knowledge need and discovery” [4]. More than 7 years after its initiation, PREDICT is still a centre wide program involving clinicians, basic scientists, leaders and core staff (nurses, care aids, therapists, physicists, radiation and medical oncologists, administrative and clerical staff), all working together to achieve a common research goal while providing cancer care.

In order to address the organizational issues faced by translational research Woolf et al. [9] indicated that adequate infrastructure is key. Zerhouni [5] noted that resources and databases that have the ability to meet the specific needs of researchers are imperative to enhance the speed of research. PREDICT also encompasses a biobank that consists of a population-based patient cohort including biological specimens, permission to contact and the ability to link to clinical outcomes data from consenting participants. By using an ethically sound governance structure in a research driven cancer facility, the PREDICT project has created the infrastructure that not only facilitates translational research but can also speed the process of addressing specific research questions. The Access Committee has become instrumental in making this process possible. Partnering with academic researchers locally, nationally and internationally, PREDICT has been able to support over 20 research projects with samples, data or permission to contact information. Through this partnership PREDICT contributes to addressing the first translational research road block: “the transfer of new understandings of disease mechanisms gained in the laboratory into the development of new methods for diagnosis, therapy, and prevention and their first testing in humans” [1,3].

Cargo and Mercer [8] identified that participation from the initiation of the research planning process was an important factor in commitment to the research process. Schmittdiel et al. [6] suggests that engaging in collaborative partnerships and investing in long-term relationships are participatory research methods that may contribute to overcoming the second translational research road blocks: “translation of results from clinical studies into everyday clinical practice and health decision making” [1,3]. Aligning with these participatory research principles, the PREDICT project successfully engaged an interdisciplinary team from the commencement of the design process. In addition, PREDICT maintains a strong presence in the clinic, with staff who are on the front-line of direct patient care involved in PREDICT governance and daily activities. We argue that our front-line approach is effective in engaging staff and consolidating a long-term relationship that contributes not only to the PREDICT platform sustainability but also may influence their engagement in other research activities. Some of the PREDICT processes such as the involvement of care-aids in triggering the consent interview are currently being adopted by other research teams in our centre.

PREDICT has also contributed to overcoming the third translational research roadblock: “the gap between knowledge need and discovery”. An empirical validation of this claim is not possible at this time however, some evidence of PREDICT bridging this gap already exists: patients with colorectal cancer contacted through the PREDICT project suggested the need for more investigation in the area of physical activity. With the support of PREDICT, a feasibility study led by investigators from our centre (testing circuit-based activities for people with colorectal cancer) was conceptualized, developed and completed. Currently a randomized clinical trial built on the results of the feasibility study and funded by a national grant is accruing participants. We recognize that formulating new research questions based on the results of a previous research project is not a new concept. What is impressive, however, is the speed of the process: within the short time-frame of three years all these activities have happened and the group estimates that a new physical activity program will be launched for all colorectal cancer patients in the clinic next year. PREDICT has been directly involved in participants’ accrual and health care providers’ engagement in the project.

### Lessons learned

The PREDICT project has evolved considerably since its conceptualization in 2006. During these seven years we have learned many lessons that had an impact on this process:

● The role of PREDICT personnel evolved as staff and processes changed in the clinic. A PREDICT assistant was responsible for obtaining written consent and collecting blood at the first stages of the project. With a new phlebotomy service being offered in the clinic, the role was modified and participants were directed to the new service after consenting.

● After changing the PREDICT assistant role to exclude phlebotomy duties, the Steering Committee identified the opportunity of training graduates from undergraduate programs to fulfill the revised role. The first PREDICT research intern was hired by the end of 2007.

● The number and role of the PREDICT research interns also changed with time and maturity of the project. It started with one person responsible for consenting and blood processing, evolved to two people each one dedicated to either blood processing or consenting, then two people sharing both tasks and now, with the changes in blood processing (discussed below), we have one person responsible for both tasks again.

● PREDICT initially processed and stored serum, plasma and buffy coat from all blood samples collected. After the first five years, storage space became limiting and funding for two interns became difficult to secure. In 2012 PREDICT started processing and storing buffy coat only in an REB approved protocol thus reducing time and storage space requirements.

● The “permission to contact” component of PREDICT became a very important and valued resource. 25% of all PREDICT applications (research studies interested in accessing PREDICT) have principally focused on this feature of the project and have not requested access to any blood sample, while others have valued both the PTC and the associated blood sample.

● The importance of the design team should not be underestimated. The group was crucial to maintain the collaborative focus of the project from the outset. Although staff has been proactive and made suggestions directly to PREDICT staff after the design team was dissolved (in 2008), we are now discussing the possibility of re-engaging the design team to address new operational issues as a way of re-emphasizing the participatory feature of PREDICT.

● The research intern is the only dedicated PREDICT staff position. All the committee(s) members have other clinical and or research duties. Although this can be viewed as an asset as PREDICT processes are part of the daily staff activities we perceive it as a challenge as well. A dedicated coordinator to support promotion of PREDICT and its availability to researchers outside our organization and to handle the gradual (and more rapid if successful with promotion) increase in number of research applications is desirable at this point. However, expanding PREDICT staff has budget implications and funding is still an issue.

### Final comments

Despite the contextualized nature of the PREDICT initiative, its model can be transferred to other out-patient clinic settings. We have created setup documentation, templates, and provide assistance to enable other centers to establish similar platforms (http://www.biobanking.org). In early 2013, PREDICT celebrated the launch of the program at the BCCA - Sindi Ahluwalia Hawkins Centre for the Southern Interior. Transferability of the model will however depend on many factors such organizational structure, leadership endorsement and funding. Nevertheless we believe that the framework developed for the PREDICT project has the potential to help scientists, clinicians and the public to overcome the translational research roadblocks.

## Competing interests

The authors declare that they have no conflicting interests.

## Author’s contributions

LB and HD assembled the data and drafted the first version of the manuscript. LB, HD and PW interpreted the data, revised critically the subsequent versions of the manuscript and have given final approval of the version to be published.

## Author’s information

LB was a PREDICT research intern between 2012 and 2013. HD is the PREDICT Senior Manager since 2008 and the Clinical Research Manager at the BCCA-VIC. PW is a pathologist, the principal investigator for the PREDICT project and has made substantial contributions to the conception and design of PREDICT.
